# *Announcement*: Click It or Ticket Campaign — May 22–June 4, 2017

**DOI:** 10.15585/mmwr.mm6619a8

**Published:** 2017-05-19

In 2015, a total of 22,441 passenger vehicle occupants died in motor vehicle crashes in the United States, representing a 6.6% increase from 2014. Among those who died, 48% were unrestrained by a seat belt (or an age- and size-appropriate car seat or booster seat for younger children) at the time of the crash, whereas only 14% of 38,152 passenger vehicle occupants who survived a crash where at least one person died were unrestrained (*1*). Using a seat belt is one of the most effective ways to prevent serious injury or death among older children, teens, and adults in the event of a crash (*2*). Despite the effectiveness of seat belts, millions of persons in the United States continue to travel unrestrained (*3*).

*Click It or Ticket* is a national campaign coordinated annually by the National Highway Traffic Safety Administration (NHTSA) to increase proper use of seat belts through safety education and strong law enforcement. *Click It or Ticket* takes place from May 22 to June 4, 2017. Law enforcement agencies across the nation will conduct intensive, high-visibility enforcement of seat belt laws, which has been demonstrated to be effective in increasing seat belt use (*4*). Enforcement is particularly encouraged from 6 p.m. until 5:59 a.m., because seat belt use is lower at night (*1*). Additional information and publication materials for the 2017 *Click It or Ticket* campaign are available from the NHTSA website at https://www.trafficsafetymarketing.gov/get-materials/seat-belts/click-it-or-ticket.

State-specific fact sheets on seat belt use, strategies to increase restraint use, and costs of crash deaths are available from CDC at https://www.cdc.gov/motorvehiclesafety/seatbelts/states.html and https://www.cdc.gov/motorvehiclesafety/statecosts/index.html. Additional information on preventing motor vehicle crash related injuries is available from CDC at https://www.cdc.gov/motorvehiclesafety.
